# Supplementary material for: Learning curve and surgical outcome of robotic assisted colorectal surgery with ERAS program
Source: Sci Rep. 2022 Nov 29;12:20566. doi: 10.1038/s41598-022-24665-w (PMC9709162; doi:10.1038/s41598-022-24665-w)
Supplement: Supplementary file 1 — Supplementary Information 1. [file 41598_2022_24665_MOESM1_ESM.zip › LSC Raw data-Ñ[▒K/100/H2_ñw╝╨░O▒Kñσ.pdf]

正式摘要(電子簽章：00000203050804)		
W55 / W55 ☐ 英文標題(注意:無英文姓名-請補病人基本檔!)	☐【讀取】憑證	

臺中榮民總醫院

出院病歷摘要

轉入號 : 轉出號 :

[redacted]

**(8)**流水編號	
**(9)**入院日期	109/11/06 (2020) 直腸外科 W55 - 283
**(10)**轉科日期	
**(11)**出院日期	109/11/12 (2020) 住院天數計 6 日

診斷

- 入院 : 1. AGE
  2. Sigmoidal cancer, s/p laparoscopic Anterior resection and anastomosis on 10/29
  3. Type II DM, under OHA
- 出院 : 1. AGE, subsided
  2. Sigmoidal cancer, s/p laparoscopic Anterior resection and anastomosis on 10/29
  3. Type II DM, under OHA

主訴

- ABD pain since the night of 2020/11/04 and vomiting in the morning of 2020/11/05.

病史

- This is a 58 y/o woman with history of 1. Sigmoidal cancer, s/p laparoscopic Anterior resection and anastomosis on 10/29 2. Type II DM, under OHA , who was discharged on 2020/11/02. She suffered from ABD pain and vomiting in the night of 2020/11/04 and she visited Yuan-Lin CH for help. Medical management was performed and the S/S subsided then she was discharged on the same day. The pain raised again and vomiting developed in the morning of 2020/11/05 and she visited our ER for help. This time, she is admitted for further care.

體檢發現

- CEA 1.3
  - General appearance :
    - easy looking , conscious clear , without cardiopulmonary distress
    - Vital signs自動帶入date 體溫(BT) 脈搏(P) 呼吸(R) 血壓(BP) 身高(BH) 體重(BW) BMI
      20201106 36C 83/min 18/min 128/89mmHg 161.5cm 65kg 24.9
  - HEENT :
    - Eyes: no anemic conjunctiva, no icteric sclera, normal EOM
    - ENT: no otorrhea, no rhinorrhea, no erythematous mucosa, no injected tonsil, no deformity
  - Neck: supple, freely movable, trachea midline,no deformity, no engorged jugular vein , no palpable thyroid or lymph node enlargement
  - Chest:
    - Inspection: Grossly normal, symmetric and fully expansion, no accessory muscle used, no operation scar, no spider angiomata, no gynecomastia
    - Palpation: no palpable mass or nodule, no subcutaneous emphysema, no tenderness
    - Ausculation: clear breathing sound
  - Heart:
    - Percussion: normal heart size
    - Ausculation: regular heart beat, normal S1, S2, no gallop, no murmur
  - Abdomen:
    - Inspection: in
      - flat contour, symetrically, no scar, no stria, no dilated veins, no rashes
    - Ausculation: normoactive bowel sound, no abdominal bruits

-
    - palpation: soft, no muscle guarding or rigidity, no local tenderness, no rebounding pain, no palpable mass, no palpable pulsation
    - Percussion: no shifting dulllness, no hepatomegaly, no splenomagaly
-

Peripheral pulsation :	CA	BA	RA	FA	PA	PTA	DPA
Right side :	++	++	++	++	++	++	++/+++
Left side :	++	++	++	++	++	++	++/+++

- Genito-anus: no tenderness or palpable mass lesion or rectal shelf, normal external genitalia, normal anal tone, brown stool

CRS CONDITION

-
    - Lung

手術日期及方法

住院治療經過

- After admission, abdominal pain was subsiding. PPN was prescribed for poor intake condition. For subsided symptoms, PPN was ceased and MBD was permitted with OPD f/u after then.

合併症

一般檢查

特殊檢查

病理報告

其它

出院時情況

- 15.計劃性出院

出院指示

-

藥名	劑量	劑量單位	用法	頻次	數量	數量單位	現況	天數	單位
Mag. Oxide tab	2	TAB	PO	TID	36	TAB	TH	6	天
Through tab	1	TAB	PO	QN	6	TAB	TH	6	天
Aescin geL 20gm		TUB	TOP	QID	0	TUB	TH	1	瓶
MOSAD tab 5mg	1	TAB	PO	TID	18	TAB	TH	6	天

出院計劃

主治醫師 2062F 蔣鋒帆 / 住院醫師 2310K 陳瑋夫

正式摘要(電子簽章：000000203050804)

W55 / W55

doctorRank:B
